# RND3 promotes Snail 1 protein degradation and inhibits glioblastoma cell migration and invasion

**DOI:** 10.18632/oncotarget.12396

**Published:** 2016-10-01

**Authors:** Baohui Liu, Huimin Dong, Xi Lin, Xiangsheng Yang, Xiaojing Yue, Jian Yang, Yuntao Li, Liquan Wu, Xiaonan Zhu, Shenqi Zhang, Daofeng Tian, Junmin Wang, Qiang Cai, Shanping Mao, Qianxue Chen, Jiang Chang

**Affiliations:** ^1^ Department of Neurological Surgery, Renmin Hospital of Wuhan University, Wuhan, Hubei, 430060, China; ^2^ Center for Translational Cancer Research, Institute of Biosciences and Technology, Texas A&M University Health Science Center, Houston, TX 77030, USA; ^3^ Department of Neurology, Renmin Hospital of Wuhan University, Wuhan, Hubei 430060, China

**Keywords:** RND3, multiform glioblastoma, snail1 signaling

## Abstract

Activation of Snail1 signaling promotes the migration and invasion of multiple tumors, including glioblastoma multiforme (GBM). However, the molecular mechanism that augments Snail1 signaling during GBM cell migration and invasion remains largely unknown. Identification of the factors that regulate Snail1 signaling is critical to block tumor cell migration and invasion. By screening human GBM specimens, we found that the expression levels of small GTPase RND3 positively correlated with the expression levels of E-cadherin and claudin, the glioblastoma migration biomarkers negatively regulated by Snail1. Downregulation of E-cadherin and claudin has been associated with the migration and invasion of GBM cells. We demonstrated that RND3 functioned as an endogenous inhibitor of the Snail-directed transcriptional regulation. RND3 physically interacted with Snail1 protein, enhanced Snail1 ubiquitination, and facilitated the protein degradation. Forced expression of RND3 inhibited Snail1 activity, which in turn blocked glioblastoma cell migration and invasion *in vitro* in cell culture and *in vivo* in GBM xenograft mice. In contrast, downregulation of RND3 augmented Snail1 activity, and subsequently decreased E-cadherin expression, eventually promoted glioblastoma cell migration and invasion. The pro-migration induced by RND3 downregulation was attenuated by Snail1 knockdown. The findings partially explain why Snail1 activity is augmented in GBM, and defines a new function of RND3 in GBM cell migration and invasion.

## INTRODUCTION

Glioblastoma multiforme (GBM, World Health Organization grade IV) is one of the most common primary tumors of the central nervous system. GBM are the highest grade of gliomas, given the characteristic abilities of high invasion, migration, and proliferation. Surgery followed by radiation and chemotherapy is the standard therapy for glioblastoma patients. Despite standard and targeted therapies, the median overall survival of GBM patients remains just over 1 year [1]. Glioblastoma migration and invasion occurs at multiple stages of cancer progression and is a clinical obstacle for therapy. Suppression of glioblastoma cell migration and invasion will provide an effective therapeutic strategy.

Snail1 is a zinc-finger transcription factor that represses E-cadherin and claudin transcription. Downregulation of E-cadherin leads to epithelial-mesenchymal transition during embryonic development, a process also exploited by invasive/migrating cancer cells [2–4]. In glioblastoma, Snail1 activity is upregulated, which promotes the tumor cell migration and invasion [5–7]. However, the mechanism of augmentation of Snail1 signaling in GBM remains largely unknown.

We recently observed that expression levels of small G protein RND3 (also called RhoE) were significantly decreased in glioblastoma patients. We also demonstrated that downregulation of RND3 promoted GBM cell proliferation and tumorigenesis [8].

RND3 is an atypical member of the Rho GTPase family in that it lacks detectable GTPase activity [9]. The well characterized functions of RND3 are its inhibitory effects on Rho kinase-mediated biological functions including actin cytoskeleton formation, phosphorylation of myosin light chain phosphatase, and cell apoptosis [10–12]. Our and other groups have revealed its Rho kinase-independent roles recently [13–18], which uncovered an indispensable role of RND3 in mouse neuron development [14, 19]. We recently showed that RND3 was highly expressed in mouse brain tissues. Genetic deletion of RND3 promoted mouse ependymal epithelia proliferation, which in turn led to aqueduct stenosis and hydrocephalus development [20]. We also demonstrated an inhibitory role of RND3 in GBM tumorigenesis through targeting Notch signaling [8]. Similar observations were detected in lung cancer and squamous cell carcinomas by other groups [21, 22], clearly indicating the important role of RND3 in tumorigenesis. In this study, we extended our previous observation, and investigated the role of RND3 in GBM cell migration and invasion. We found that RND3 deficiency facilitated GBM cell migration and invasion, and revealed the associated molecular mechanism mediated by RND3.

In this study, we provided evidence that that RND3 inhibited GBM cell migration and invasion partially through inhibiting Snail1 activity. Forced expression of RND3 diminished Snail1 activity, which in turn impeded glioblastoma cell migration and invasion. Downregulation of RND3, however, enhanced Snail1 signaling, in which repressed E-cadherin and claudin expressions, therefore facilitated glioblastoma cell migration and invasion. Furthermore, we uncovered the molecular mechanism of the RND3-mediated Snail1 inhibition. RND3 physically interacted with Snail1, and promoted the protein degradation. The study characterized a new function of RND3 in GBM migration and invasion, and provided a new insight into the inhibitory effect of RND3 on Snail1 regulation and Snail-mediated GBM cell migration and invasion.

## RESULTS

### Significant downregulation of E-cadherin, claudin and RND3 protein levels were detected in human GBM tissues

**Figure 1:**
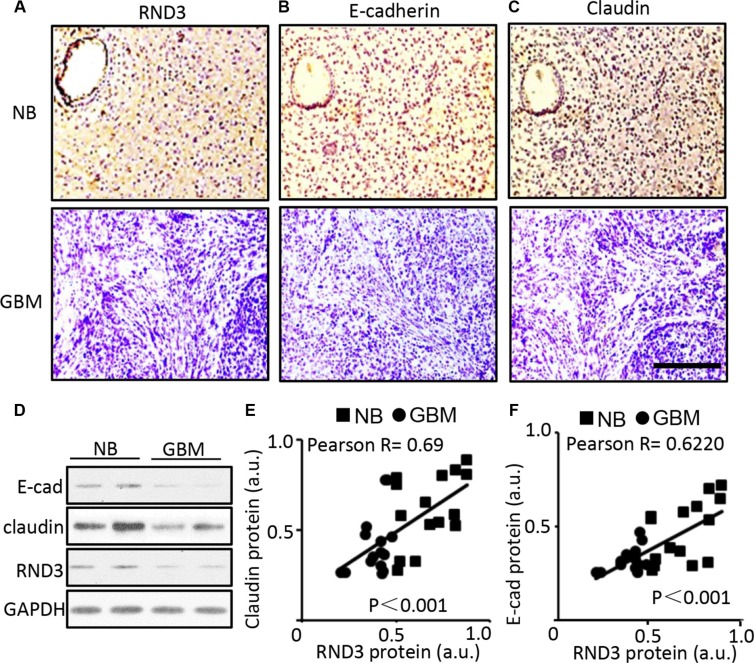
Significant downregulation of E-cadherin, claudin and RND3 protein levels were detected in human GBM tissues (**A–C)** Representative immunohisochemical staining (brown) for RND3, E-cadherin, and claudin in human glioblastoma tissues serial sections, respectively. Blue color indicates nuclear staining. (**D**) Immunoblotting analysis showed the expression levels of RND3 and E-cadherin, claudin in human normal brain (NB) and glioblastoma (GBM). (**E–F)** Quantification of the immunohistochemical staining for RND3 and E-cadherin and claudin showed a positive correlation of the two protein expression levels in human GBM tissues (*n* = 15) and brain tissues (*n* = 15). Statistical analysis of correlation was performed with Pearson's test. Scale bar: 10 μm. a.u.: arbitrary unit.

To determine the clinical significance of RND3 in gliomas, we assessed and compared the expression levels of RND3 transcript and protein in human GBM specimens, the normal brain tissues (NB) (glioma adjacent brain areas, 3 cm away from glioma). To explore the relationship between RND3 expression and glioblastoma migration/invasion, we assessed the expression levels of RND3 along with two GBM migration biomarkers E-cadherin and claudin in human GBM tissues and normal brain tissues [23–26]. All of the assessments were performed by immunohistochemical analyses (IHC) and Western blot. In human glioblastoma tissues serial sections from normal brain tissue to GBM tissue, significant decreases in RND3, E-cadherin and claudin protein levels were observed in human GBM tissues compared to normal brain tissues (Figure 1A–1D). The correlation of the changes of RND3 versus E-cadherin and claudin were analyzed by Pearson's correlation coefficient. The result suggested a strong positive correlation between the changes of RND3 protein levels and E-cadherin and claudin protein levels (Figure 1E and 1F) in human GBM specimen.

### RND3 inhibited glioblastoma cell migration and invasion *in vitro* and *in vivo*

**Figure 2:**
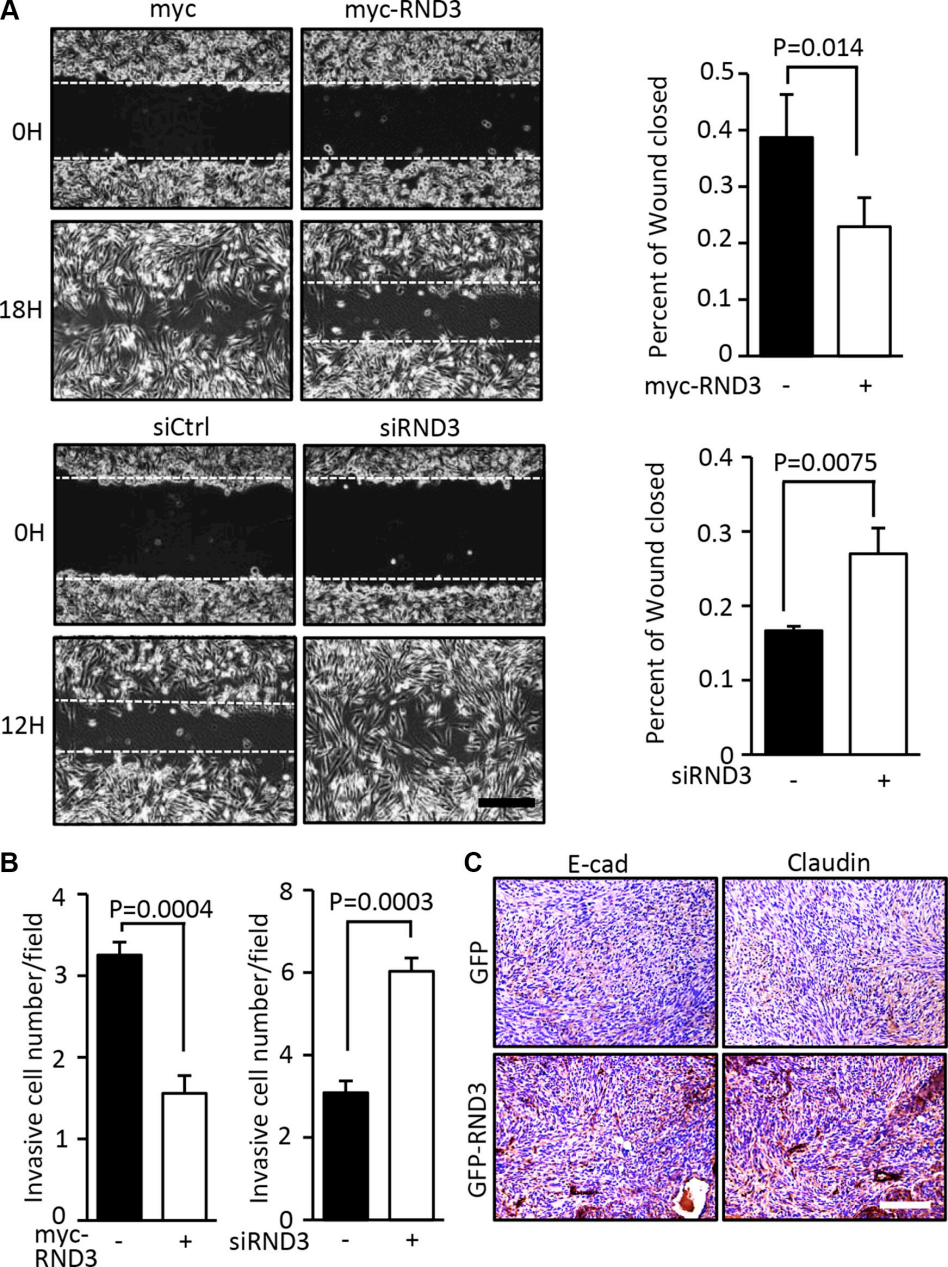
Forced expression of RND3 inhibited glioblastoma cell migration and invasion (**A–B)** U251 cells, a human GBM cell line, were transfected with myc-RND3 or siRND3 to overexpress or knockdown RND3, respectively. Transfected cells were synchronized and cultured in the growth medium to assess the migration and invasion by wound healing and transwell assay, respectively. Migration /invasion of U251 cells were repressed after exogenous RND3 was introduced, while treatment of siRND3 promoted invasion. In right side of panel A, the closure of wound gap was quantified from nine images. In panel B, each experiment was duplicated and motile cells were counted from 20 fields of each experiment (10 field/per duplicates). (**C)** *In vivo* E-cadherin and claudin expression in tumors were assessed by immunohistochemical staining (dark red). Tumors generated from nude mice by intracranial implantation of GFP-RND3 cells, a U251 cell lines with GFP-RND3 stable expression, were compared to the tumors derived from the control mice with intracranial implantation of U251 cells expressing GFP. Statistical analysis was performed with the Student's *t* test in A and paired Student's *t* test in B. Scale bar represents 100 μm in A and C.

To evaluate the effect of RND3 on GBM cell migration, wound healing assay and Transwell migration assay were conducted in human GBM U251 cells with overexpression and downregulation of RND3, respectively. In the wound healing assay, cell migration was significantly impeded in the U251 cells with RND3 overexpression, while cell migration was promoted in the RND3-knockdown U251 cells (Figure 2A, Supplementary Figure S1). Consistently, quantitative analysis of Transwell invasion assays confirmed the wound healing results (Figure 2B), in which forced expression of RND3 prevented cells from crossing the well and knockdown of RND3 facilitated the invasion process.

The positive regulation of RND3 in E-cadherin and claudin expression during GBM development was demonstrated *in vivo* by generating a human orthotopic GBM xenograft animal model. The experiment was conducted by intracranial implantation of U251 glioblastoma cells with stable RND3 expression in nude mice. Significant increases in the expression levels of both E-cadherin and claudin were observed in the tumor developed from the RND3 stable expression U251 cells.

### Expression of E-cadherin was closely regulated by RND3

**Figure 3:**
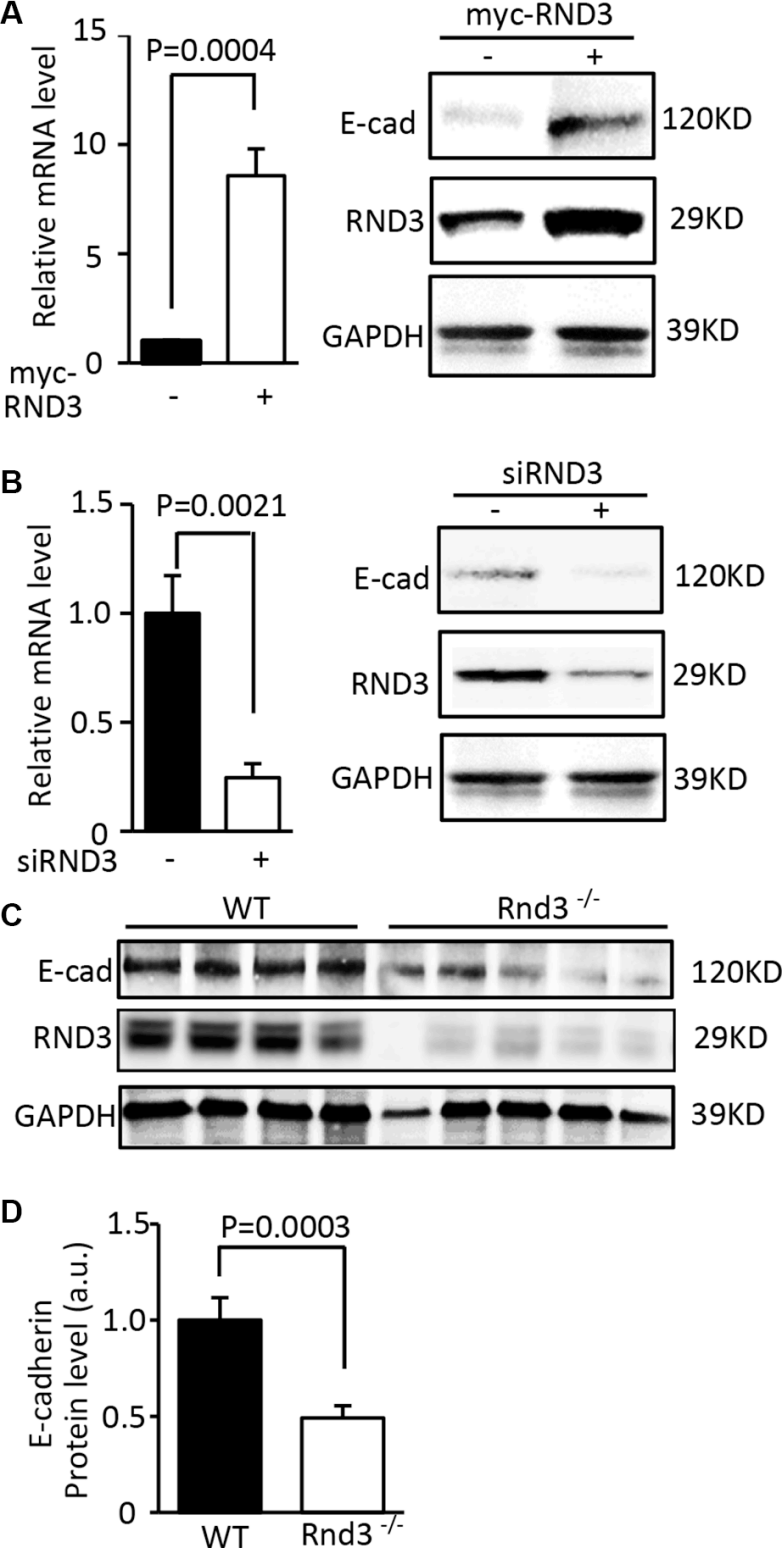
Expression of E-cadherin was closely regulated by RND3 (**A**) Forced expression of RND 3 upregulated EMT marker E-cadherin transcript (left panel) and protein levels (right panel) in U251 cells. (**B**) Knockdown of RND 3 resulted in inhibition of E-cadherin transcript (left panel) and protein levels (right panel) in U251 cells. (**C**) Protein expression level of E-cadherin was significantly decreased in Rnd3-null mouse brain tissues compared to the WT control mice. (**D**) This downregulation was quantified. Student's *t* test was used.

E-cadherin is a critical factor involved in GBM cell migration/invasion. Downregulation of E-cadherin is necessary for tumor cell migration. We, therefore, evaluated the significance of RND3 on E-cadherin expression. The protein and mRNA levels of E-cadherin were measured in U251 cells with overexpression and downregulation of RND3. As shown in Figure 3A, forced expression of RND3 resulted in significant increases in E-cadherin protein and transcript. Meanwhile, downregulation of E-cadherin was detected in RND3 knockdown cells (Figure 3B). Same results were observed in human GBM cell line U87 (Supplementary Figure S2). To evaluate the effect of RND3 on E-cadherin expression *in vivo*, we analyzed the expression levels of E-cadherin in Rnd3 null mouse brain. The Rnd3 knockout mice were generated and reported in our previous study [13]. Again, a less than half of E-cadherin protein levels were found in Rnd3 null mouse brains compared to wild-type control mouse brains (Figure 3C and 3D).

### RND3 expression levels were inversely correlated with Snail1 expression levels in human and mouse GBM tissues

**Figure 4:**
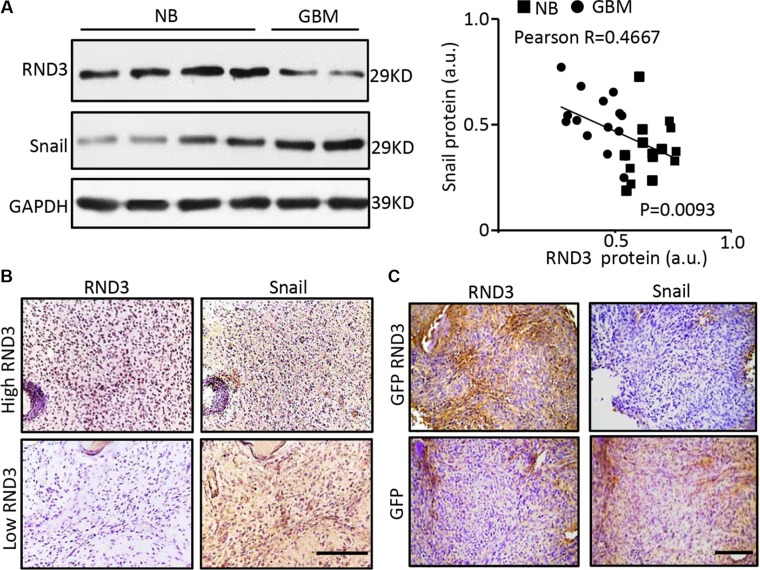
RND3 expression levels were inversely correlated with Snail1 expression levels in human and mouse GBM tissues (**A–B)** the protein levels of RND3 and Snail1 in human GBM tissues are detected by immunoblotting and immunohistochemical staining (brown). Quantification of the immunoblotting for RND3 and Snail showed an inverse correlation of the two protein expressions in human normal brain (NB) and glioblastoma (GBM) tissues (*n* = 30) (A, right panel). (**C)** Significant decrease in snail expression was shown in the GBM xenograft nude mouse brains with intracranial implantation of U251 cells overexpressing GFP-RND3 compared with the control mice with the implantation of U251 cells expressing GFP only. Pearson's test was performed for the correlation analysis. Scale bars represent 100 μm.

It is well known that Snail is one of the major transcriptional repressors to inhibit E-cadherin expression [27], and Notch can upregulate Snail signaling [28]. Our previous study found that Notch signaling was significantly enhanced in Rnd3 deficient mouse brain [13]. We, therefore, investigated of the correlation between RND3 and Snail1 towards clinical evaluation of thirty human GBM specimens. The expression levels of RND3 and Snail1 protein in GBM tissues were assessed by Western blots (Figure 4A). The data indicated a significant increase in Snail protein expression along with a downregulated Rnd3 protein level in the human GBM tissues, strongly suggesting an inverse correlation between the two factors with −0.4667 Pearson product-moment correlation coefficient (Figure 4A, right panel). The increase in Snail expression along with the decrease in Rnd3 expression level was confirmed and visualized by immunostaining in human GBM (Figure 4B) and the GBM xenograft nude mice (Figure 4C).

### Inhibition of Snail1 curtailed RND3 deficiency-induced GBM migration and invasion promotion

**Figure 5:**
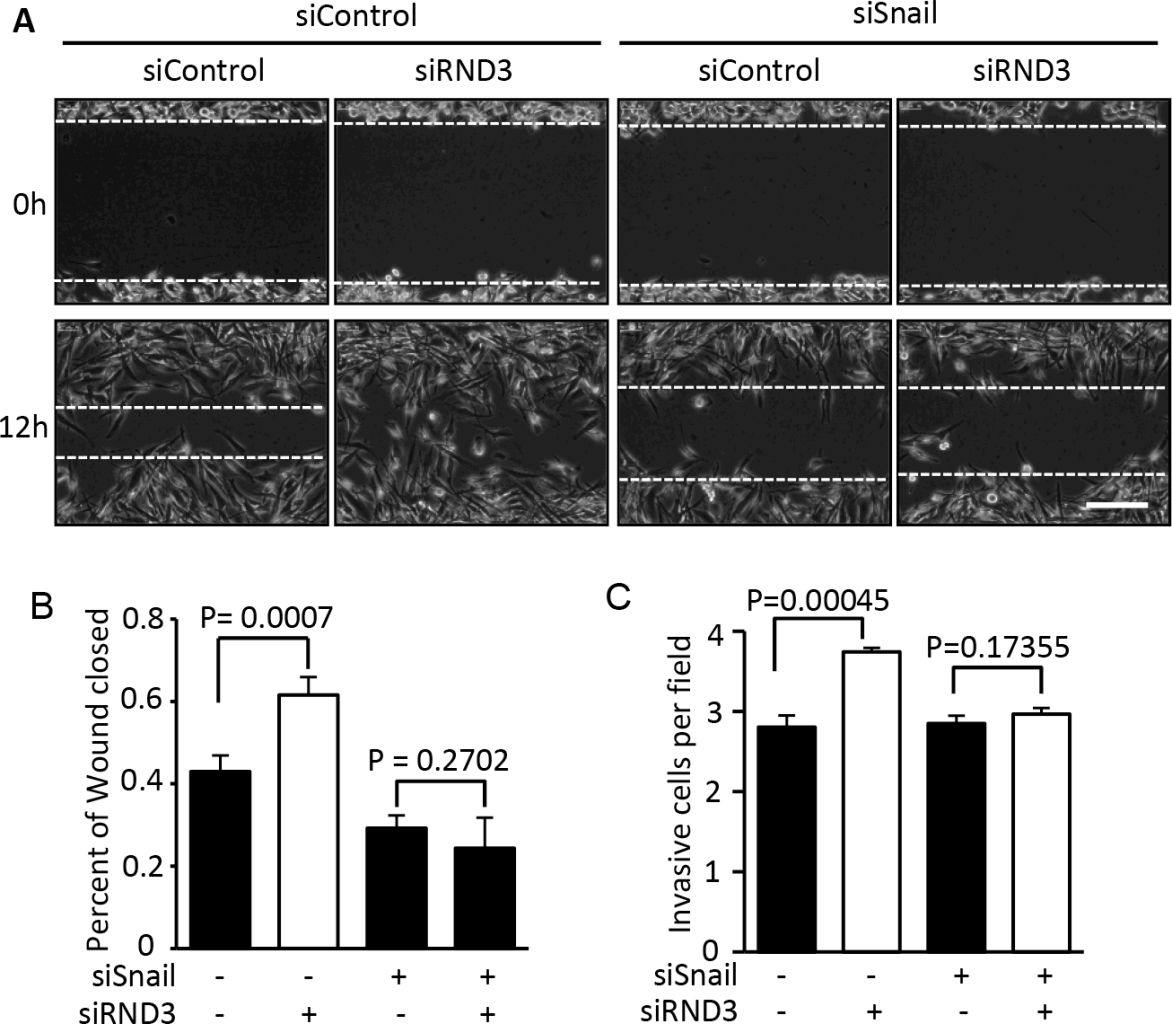
Knockdown of Snail1 diminished the promotion of RND3 deficiency-induced GBM cell migration and invasion The migration and invasiob of GBM cells was assessed by wound healing and transwell assay, respectively. (**A–B**) The wound healing experiment showed that RND3 deficiency promoted tumor cell migration. This enhancement of the cell migration was completely blocked by Snail knockdown. (**C**) A similar result was achieved in a transwell assay. Student's *t* test was used for the statistical analyses. Scale bar represents 100 μm.

To investigate if RND3 deficiency promotes GBM cell migration and invasion through a Snail1-dependent signaling mechanism, we introduced short interfering RNA (siRNA) specific for Snail1 and RND3, respectively. Wound healing and Transwell migration assays were performed in the Snail1 and RND3 knock down U251 cells. As expected, knockdown of Snail1 inhibited cell migration in wound healing and invasion in Transwell assays. Oppositely, downregulation of RND3 significantly accelerated cell migration and invasion. Moreover, this RND3 deficiency-induced cell migration and invasion facilitation was completely diminished by Snail1 knockdown (Figure 5A–5C). The results suggest that Snail1 is responsible for RND3 deficiency-induced GBM cell migration and invasion promotion.

### RND3 enhanced Snail1 protein degradation

**Figure 6:**
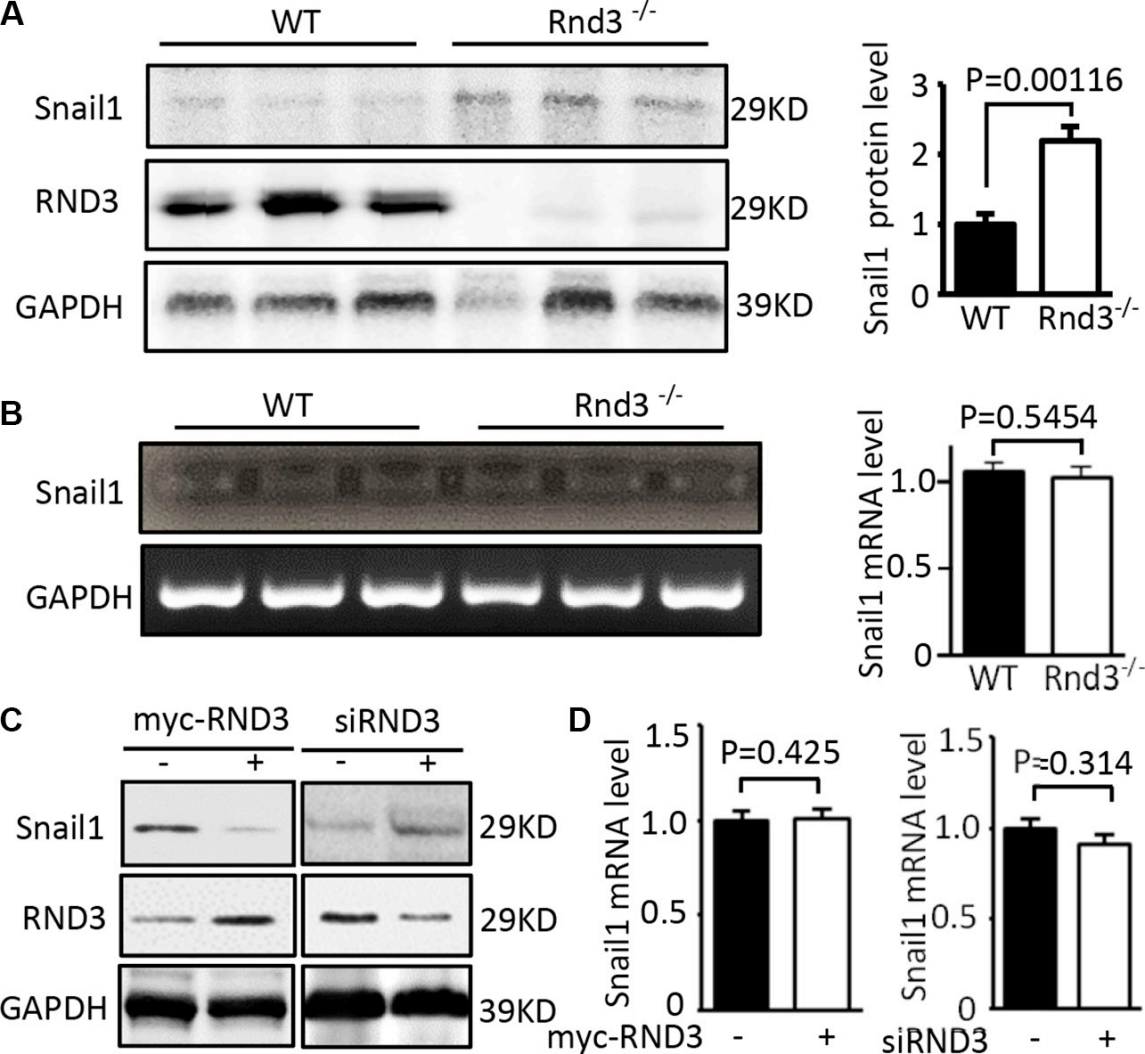
RND3 negatively regulated the expression levels of Snail protein but not the transcript (**A**) Immunoblot analysis showed that Rnd3 deficiency resulted in increases in the Snail at protein levels in Rnd3 null mouse brain tissues. (**B**) No changes of Snail1 transcripts were found in the same cohort of Rnd3 null mouse brains. (**C**) Forced expression of RND3 in U251 cells resulted in a decrease in Snail1 protein level. An opposite result was observed when RND3 was knocked down. (**D**) The transcript levels of Snail1 remained unchanged under overexpression and downregulation of RND3 conditions. Student's t was used for the statistical analyses.

In the same cohort of Rnd3 null mice used for the assessment of E-cadherin, we measured Snail1 protein and transcript levels. The expression level of Snail1 protein was significantly increased in Rnd3 null mouse brains compared with wild-type control mouse brains (Figure 6A). However, no significant change of Snail transcript was observed in the Rnd3 null brains (Figure 6B). The same results were achieved *in vitro* in cell culture studies by knocking down RND3 using siRND3 in U251 cells. In contrast, forced expression of RND3 decreased Snail1 protein expression level without the change of Snail1 transcript level (Figure 6C and 6D). These data suggest that Snail1 is under post-translational regulation promoted by Rnd3.

### RND3 physically interacted with Snail1 and promoted Snail1 degradation through the protein ubiquitination

**Figure 7:**
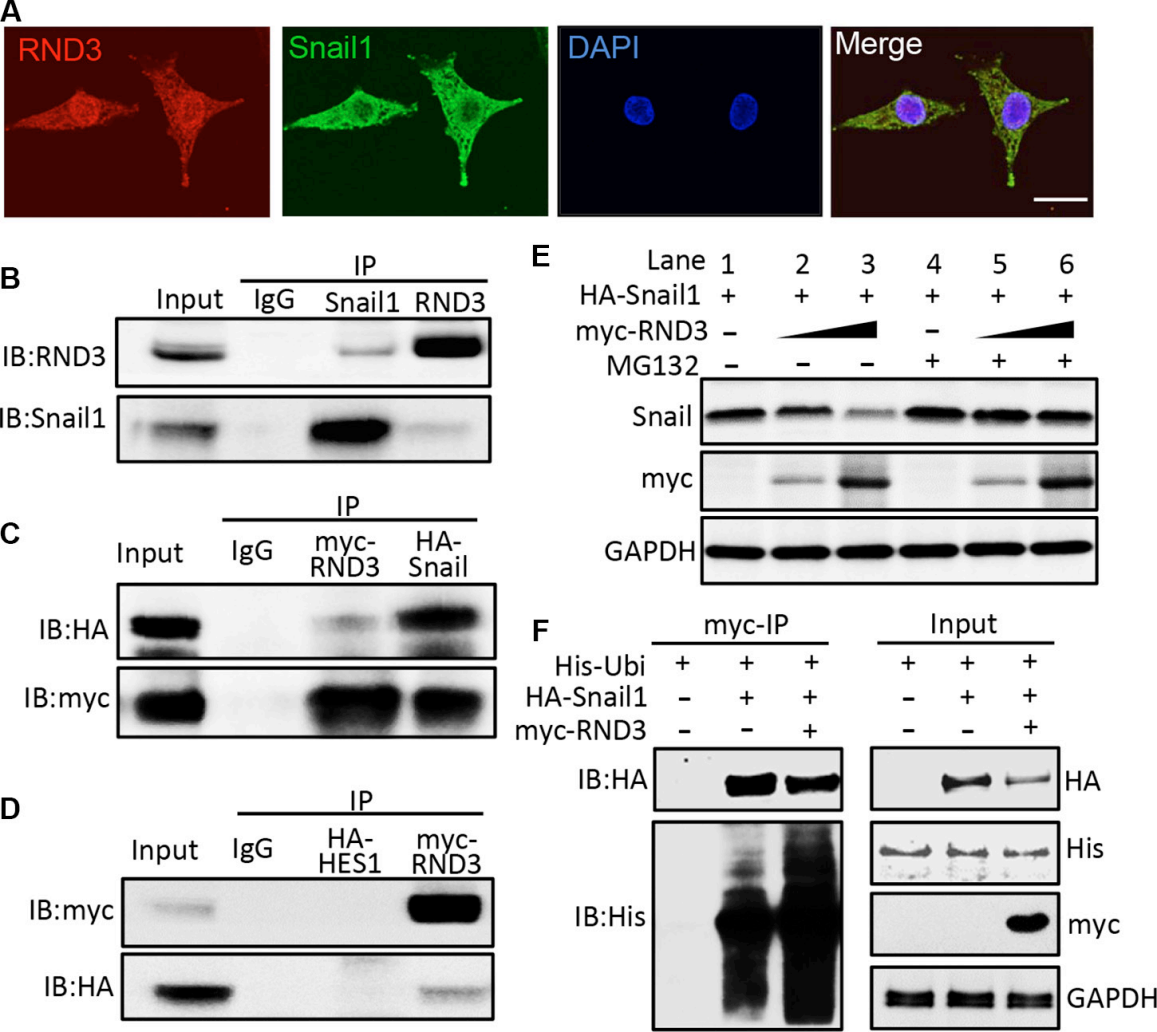
Rnd3 physically interacted with Snail1 and facilitated Snail1 protein degradation (**A**) immunofluorescent staining displayed the colocalization of RND3 (green) and Snail1 (red) expression in U251 cells. (**B–D**) Co-immunoprecipitation (IP) pull-downs were conducted at both endogenous (B) and exogenous level (2 μg of each plasmid was co-transfected) (C) followed by immunoblotting analysis (IB). The blots demonstrated the interaction of RND3 and Snail1 proteins while no non-specific binding with negative control, HA-HES1 (D). (**E**) The Snail1 protein levels in the whole cell lysates transfected with the RND3 expression vector were assessed by immunoblotting analysis. Along with the elevations of RND3 protein levels, noticeable decreases in the Snail1 protein levels were observed and this effect was partially attenuated by treatment with a proteasome inhibitor MG-132(10 μM). (**F**) A representative immunoblotting analysis for ubiquitin showed the increase in the ubiquitination level of Snail1 pull-down complex in the forced expression of RND3 group. Meanwhile, the reduction in Snail1 protein levels was detected in parallel with this enhanced ubiquitination. Scale bar represents 50 *μ*m.

Snail protein is tightly regulated by ubiquitin-proteasome system (UPS). Ubiquitinated Snail is quickly degraded by the proteasome. We investigated whether RND3 negatively regulates post-transcription of Snail1 protein by promoting UPS-mediated degradation of Snail1 protein. We checked the expression profiles of the two proteins by immunofluorescent staining, and found that RND3 and Snail1 were co-localized in the U251 glioblastoma cells (Figure 7A). We then performed co-immunoprecipitation mutual pull-down assays at endogenous levels, and at exogenous levels by co- transfection of myc-RND3 and HA-Snail1 in U251 cells. The results demonstrated the existence of a physical interaction between RND3 and Snail1 in GBM cells (Figure 7B–7D).

To understand the biological significance of the interaction between the two proteins, the levels of Snail1 protein were assessed along after the increase in RND3 expression. Immunoblot analysis showed a decline in Snail1 protein levels when the cells expressed higher levels of RND3 (Figure 7E, lanes 1–3). However, this trend was remarkably attenuated by the treatment with proteasome inhibitor MG132 (Figure 7E, lanes 4–6).

In the same experiment, the levels of Snail1 ubiquitination were analyzed by immunoprecipitation of Snail1 followed by an anti-ubiquitin immunoblot analysis. As shown in Figure 7F (lower panel in the myc-IP section), the amount of ubiquitinated species was clearly increased after the introduction of RND3. In parallel with the increase in Snail1 ubiquitination, lower expression levels of Snail1 protein were detected in the same pull-down sample (Figure 7F, upper panel in the myc-IP section). These data indicate that RND3 physically interacts with Snail1, and facilitates UPS-mediated Snail1 protein degradation.

## DISCUSSION

The direct and mechanistic role of RND3 in GBM migration and invasion remains largely unexplored. The result of this study show that RND3 directly regulate Snail stability through physically interact with it in GBM cell. These finding demonstrate that RND3 can effectively exert its control over the GBM cell migration and invasive.

The first line of treatment for glioblastoma involves surgical resection, followed by rounds of radiotherapy and chemotherapy. However, this treatment fails in the majority of cases largely owing to the invasive/migratory nature of the tumor [29]. The limitation of resection, radiotherapy, and chemotherapy is the inability of the treatment to remove all tumor cells. Owing to the migration of cells, and to avoid damage to healthy tissue, the current treatment misses many of the tumor cells in the brain. In cases of recurrence, anti-angiogenic therapeutics are applied to prolong life, but this treatment rarely offers a cure [30, 31]. Therefore, our finding in this study, suppression of GBM cell migration and invasion by RND3 through repressing Snail, is a critical need as new molecular targets, alternative concepts, and approaches to treat this devastating disease.

Tumor migration is controlled through many signaling pathways, including Snail1 signaling. It is well established that Snail1 signaling is crucial for tumor migration. Sailer et al. previously reported that Snail1 was upregulated in seven samples of GBM compared with normal human brain tissues [7]. In our study, we also confirmed his finding in normal brain and GBM tissue from human patients. In terms of repression of Snail1 signaling, it was observed that transforming growth factor-beta (TGFβ) affected the increased migration and invasion of human prostate cancer cells with otherwise high Snail1 activity [32]. Parnate, an enzymatic inhibitor of Snail1, can suppress the motility and invasiveness of cancer cells with different origins and genetic backgrounds [1]. In human hepatocellular carcinoma (HCC) cells, heterogeneous nuclear ribonucleoprotein AB (HNRNPAB) overexpression promotes cell migration and invasion through transcriptional activation of Snail1 [33]. In our study, we provide solid evidence that RND3 is also endogenous repressor for Snail signal in GBM migration and invasion.

**Figure 8:**
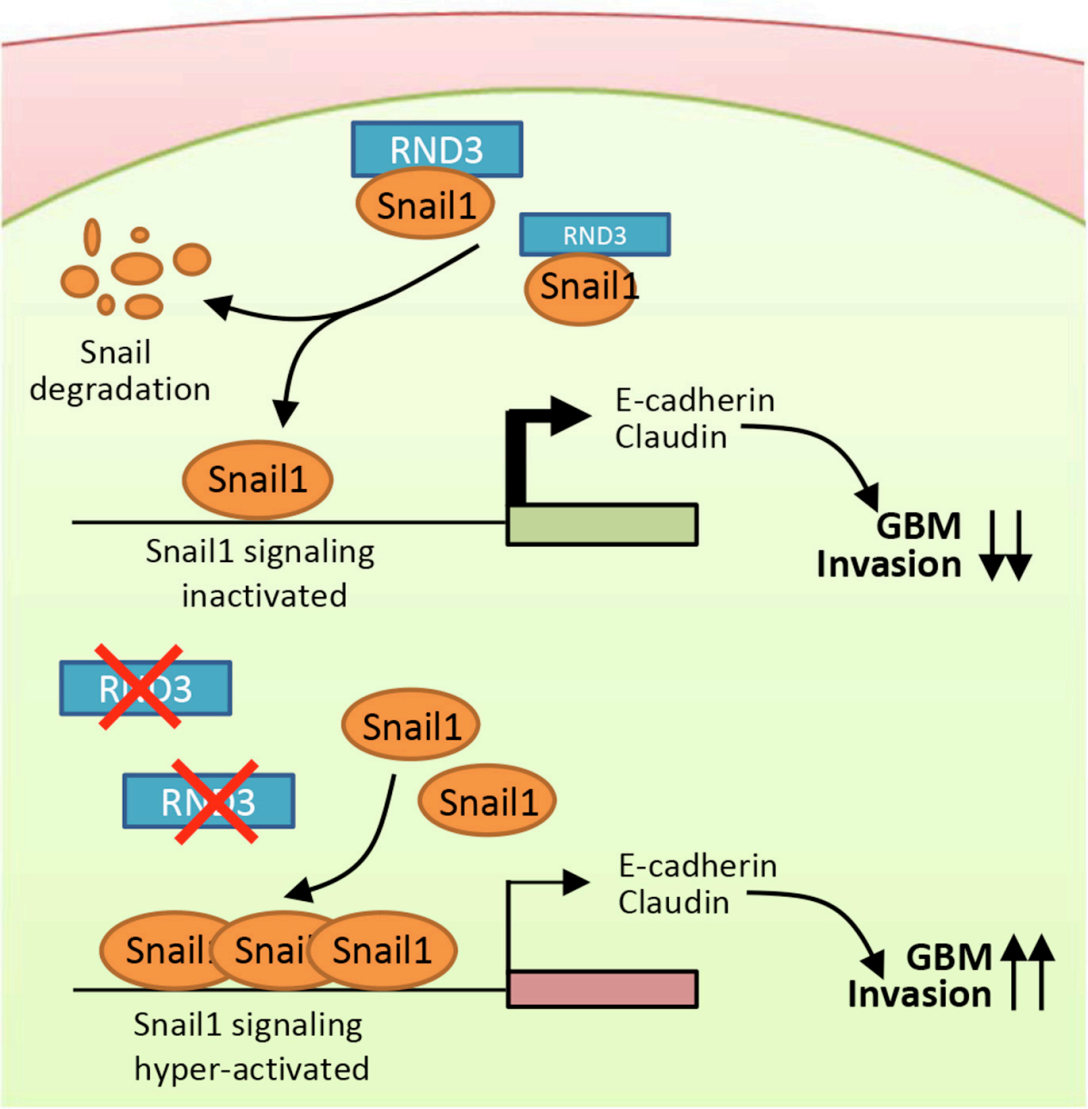
A proposed model outlining the molecular mechanism of the Rnd3 negatively regulating GBM cell migration/invasion through repressing snail signaling In the presence of RND3, RND3 represses Snail activity by physically interacting with Snail protein and promoting its degradation. In the absence of RND3, the snail signaling is significantly enhanced due to the extra amount of Snail proteins available, which represses E-cadherin and claudin expressions, promoting tumor cell migration and invasion.

In previous studies, the mechanism of RND3-mediated regulation in cell migration was mainly focused on the inhibition of Rho kinase signaling pathways. However, emerging evidence has suggested that other regulatory signaling pathways are also involved in RND3-mediated regulations. In esophageal squamous cells, forced expression of RND3 suppresses cell migration through modulation of the PTEN/PI3K/Akt signaling pathway [34]. In gastric cancer, RND3 promotes metastasis by enhancing expression of CXCR4 [35]. In HCC cells, siRNA-mediated downregulation of RND3 expression resulted in a loss of E-cadherin at cell-cell junctions [36]. The HCC study is consistent with our results, which the repressed Snail1 expression along with the consequent upregulated E-cadherin expression were achieved by RND3 overexpression in GBM cells, supporting our hypothesis [37, 38]. However, previous studies in different tumor cells, how the RND3 repress the snail activity remains largely unknown.In addition to this evidence, our study provides evidence that RND3 is a novel repressor of Snail1 signaling and glioblastoma migration and invasion. RND3 regulates Snail1 activity through post-translational regulation of Snail1. RND3 physically interacts with Snail1 and facilitates Snail1 protein degradation. We also propose a working model in which RND3 functions as a Snail1 cofactor (Figure 8). In this model, RND3 binds to Snail1 and prevents Snail1 from forming a dynamic functional complex in the nucleus. Downregulation of RND3 promotes Snail1, which then allows for the formation of the related regulatory complex and activation of Snail1-mediated transcription. Consequently, up-regulated Snail1 signaling promotes GBM cell migration and invasion.

In summary, we demonstrated that RND3 negatively regulate glioblastoma migration and invasion through a Snail1-dependent pathway. We found that RND3 is a novel repressor of Snail1. RND3 can facilitate Snail1 degradation through the UPS, a post-translational regulation mechanism rather than transcriptional regulation of Snail1. Deficiency of RND3 attenuates Snail1 protein degradation, resulting in an increase in Snail1 signaling activity, which in turn promotes cell migration and invasion. Our finding of RND3′s effect on Snail1 adds a new regulatory layer to Snail1 signaling. Given the fundamental role of the Snail pathway in cell invasion/migration, the present finding provides a potential new target for glioblastoma therapy.

## MATERIALS AND METHODS

### Human GBM samples and control brain tissues

Human GBM (grade IV) tissues were obtained at the time of surgery from the Department of Neurosurgery and Neurology in Renmin Hospital of Wuhan University. Control adult human brain tissues were collected from unmatched patients undergoing surgery for intracranial hypertension. Diagnosis by tumor histology was confirmed independently by two neuropathologists. The procurement and use of tissue for the study were performed with written patient-informed consent and approval by the Institutional Ethics Committee of the Faculty of Medicine at Renmin Hospital of Wuhan University (approval number: 2012LKSZ (010) H).

### Generation of the human GBM xenograft mouse model

Congenitally athymic male nude mice were used in the study at 5–8 weeks old (Charles River Laboratories, Wilmington, MA, USA). Intracranial injection of U251 cells into the lateral ventricle was performed as described previously [39]. A total concentration of 5 × 10^5^ cells in 3 μL of PBS, or PBS alone for the control, was injected slowly into each of the mice. Tumor growth was analyzed 15 weeks after the injection. All experiments with animals were approved by the Institutional Animal Care and Use Committee of the Texas A&M University Health Science Center in Houston.

### Cell culture, gene transient transfection, and generation of stable cell lines

Human malignant glioma (U251) cells were cultured in Dulbecco's modified Eagle's medium with 10% FBS. All of the gene transient transfections were conducted by the NEON transfection system (MPK5000; Life Technologies).

SiRNAs specific for RND3 and Snail1 were purchased from Thermo Scientific Dharmacon RNAi Technologies (RND3: L-007794-00-0005; Snail1: M-010847-00, Lafayette, CO, USA). Construction of myc-RND3 overexpression vectors was done as previously described [20]. HA-Snail1 and HA-Ubiquitin expression vectors were from Addgene.

To establish the RND3 knockdown stable cell line and RND3 constitutive expression stable cell line, lentiviral vectors V3LHS_346799 (Thermo Scientific) expressing short hairpin RNAs (shRNA) specific for human RND3, and pLVX-AcGFP1-C1 Vectors (632155, Clontech) each with an insertion of human RND3 cDNA, were used, respectively, in U251 cells. Vectors expressing non-silencing shRNA (RHS4346), and pLVX-AcGFP1-C1 vectors without RND3 cDNA insertion, were used, respectively, as controls. Briefly, 293FT cells (Invitrogen, Carlsbad, CA, USA) were transfected with the lentiviral vector expressing specific shRNA and with two helper vectors, pMD2.G and psPAX2, to produce the lentivirus. Cells were infected with the lentivirus at a multiplicity of infection of 10 with polybrene (8 g/mL) to enhance the virus transduction. The efficiency of viral infection was monitored by GFP expression. Puromycin (10 g/mL) was used for the cell selection [40].

### Wound healing assays

U251MG cells were seeded in 6-well plates and cultured until they reached confluence. A wound was then created by manually scraping the cell monolayer with a 200-μL pipette tip. The cultures were washed twice with SFM to remove any floating cells. The cells were then incubated in Dulbecco's modified Eagle's medium supplemented with 1% FBS. Cell migration into the wound was observed at fourpreselected time points (0, 12, 18, 24 h) in eight randomly selected microscopic fields for each condition and time point. The distance traveled by the cells was determined by measuring the wound width at different time points and then subtracting each measurement from the wound width at time 0. The values obtained were expressed as migration percentages, setting the gap width at 0 h as 0%.

### Transwell assays

Cells (4 × 10^5^) were plated either on the top side of polycarbonate Transwell filters without Matrigel in the case of the Transwell assay, or plated on the top side of polycarbonate Transwell filters coated with Matrigel for the Transwell matrix penetration assay, in the top chamber of the QCM 24-Well Cell Invasion Assay (Cell Biolabs, Inc.). For Transwell invasion assays, cells were suspended in medium without serum, and medium supplemented with serum was used as a chemoattractant in the bottom chamber. The cells were incubated at 37°C for 12 h. The non-migratory or non-invasive cells in the top chambers were removed with cotton swabs. The migrated cells on the lower membrane surface were fixed in 100% methanol for 10 min, air dried, and then stained with 4′, 6-diamidino-2-phenylindole and counted under a microscope. Three independent experiments were conducted and the data were presented as the means ± standard errors of the means.

### Quantitative PCR analysis

Messenger RNAs were quantified using quantitative PCR analysis (Applied Biosystems StepOnePlus) and the SYBR green method, with a MasterMix buffer system containing Taq polymerase (Stratagene), as has been described previously [41]. Total RNA was prepared by TRIzol extraction (Gibco BRL). The forward and reverse PCR primers (5′ to 3′) were as follows: RND3: CTATGACCAGGGGGCAAATA/TCTTCGCTTTGTCCT TTCGT; Snail1: GCGAGCTGCAGGACTCTAAT / CCTC ATCTGACAGGGAGGTC, E-cadherin; TGAAGGTGA CAGAGCCTCTGGAT/TGGGTGAATTCGGGC TTGTT; claudin: CACGTTCGACCAATGC/CCCGTTCCATAGG CTC; GAPDH: GAGTCAACGGATTTGGTCGT/TTGA TTTTGGAGGGATCTCG. GAPDH expression levels were used for quantitative PCR normalization. Expression levels were determined by the 2^−ΔΔCt^ threshold cycle method.

### Immunostaining, immunoblotting, and immunoprecipitation

The following antibodies were used for immuno-analyses: anti-RND3 (Cocalico Biologicals), anti-Snail1 (Santa Cruz, sc-28199), anti-E-cadherin (CST, 3195S), anti-claudin (Bioword, BS1063), anti-c-myc (9E10, Santa Cruz, sc-40), and anti-HA (Santa Cruz, SC-7392, SC-805). Even protein loading in the immunoblotting analysis was verified by the intensity of the GAPDH blot (Santa Cruz, sc-20357). The immunostaining, immunoblotting, and immunoprecipitation were conducted as described previously [8, 20]. The immunoblotting densitometry was quantified by the Gel Logic 6000 PRO Imaging System (Carestream Health, Inc. Rochester, NY, USA), and the immunofluorescent and immunohistochemical image quantifications were conducted by Leica Application Suite Imaging Software (Version 4.0, Biberach, Germany).

### Statistical analyses

Data were expressed as means ± standard errors of the means. Statistical analyses were performed with SigmaPlot version 11.0 and SPSS version 13.0. Differences between means were assessed with the Student's *t* test, paired *t*-test, or Mann–Whitney *U* test in cases of abnormally distributed data. In multiple comparisons, one-way analysis of variance was used. Pearson's test was used to detect the correction of two groups and compare quantitative values of expression. A value of *P* < 0.05 was considered statistically significant.

## SUPPLEMENTARY MATERIALS


