# Supplementary material for: RND3 promotes Snail 1 protein degradation and inhibits glioblastoma cell migration and invasion
Source: Oncotarget. 2016 Oct 1;7(50):82411–23. doi: 10.18632/oncotarget.12396 (PMC5347701; doi:10.18632/oncotarget.12396)
Supplement: Supplementary file 1 [file oncotarget-07-82411-s001.pdf]

RND3 promotes Snail 1 protein degradation and inhibits glioblastoma cell migration and invasion

Supplementary Materials

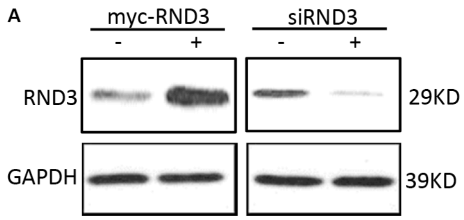

Supplementary Figure S1: Forced expression of RND3 inhibited glioblastoma cell migration and invasion. Immunoblot analysis showed significant increase of RND3 level after forced expression of RND3 and downregulation of RND3 after siRND3 treatment.

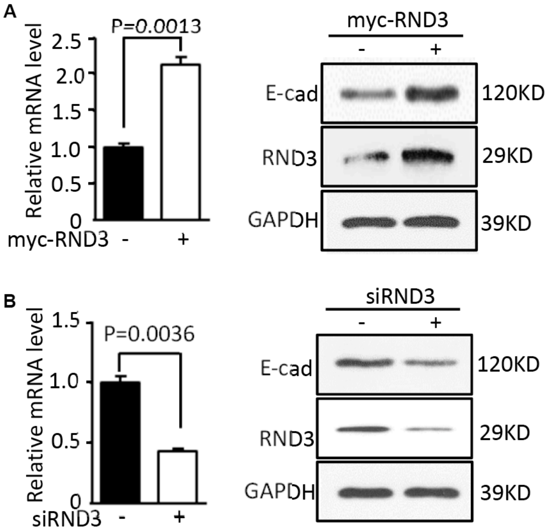

Supplementary Figure S2: RND3 also positively regulated E-cadherin expression in U87 cells. (**A**) Forced expression of RND 3 in U87 cells upregulated E-cadherin transcript (left panel) and protein levels (right panel). (**B**) Downregulation of RND3 by siRNA treatment also led to repression of E-cadherin transcript (left panel) and protein levels (right panel) in U87 cells.
